# Bioprosthetic or native aortic scallop intentional laceration to prevent coronary artery obstruction using available devices in Japan

**DOI:** 10.1007/s12928-022-00899-3

**Published:** 2022-11-24

**Authors:** Masaki Nakashima, Yusuke Enta, Kazunori Ishii, Masaki Miyasaka, Masaki Hata, Norio Tada

**Affiliations:** 1 Department of Cardiology, Sendai Kosei Hospital, 4-15 Hirose-Machi, Aoba Ward, Sendai, Miyagi 980-0873 Japan; 2 Department of Laboratory Medicine, The Jikei University School of Medicine, 3-25-8, Nishi-Shimbashi, Minato-ku, Tokyo 105-8461 Japan; 3 Department of Cardiovascular Surgery, Sendai Kousei Hospital, Sendai, 4-15 Hirose-Machi, Aoba Ward, Miyagi 980-0873 Japan

A technique termed as “bioprosthetic or native aortic scallop intentional laceration to prevent coronary artery obstruction (BASILICA)” has been developed to prevent coronary obstruction (CO) during transcatheter aortic valve implantation (TAVI). However, one reason hindering the use of this technique in Japan is the unavailability of some mandatory devices including Astato XS 20 300-cm guide wire (Asahi Intecc) and PiggyBack^®^ Wire Converter (Vascular Solutions), which is a locking, hubless microcatheter serving to insulate the guidewire shaft [1].

A 78-year-old female presented with dyspnea on exertion and was diagnosed with prosthetic valve stenosis (a mean pressure gradient of 84 mmHg, effective orifice area of 0.51 cm^2^) due to structural valve deterioration of a 19-mm Trifecta bioprosthesis (Abbott). Our heart team concluded that she was unsuitable for an open-heart repeat surgery due to her Society of Thoracic Surgeons (STS) score of 21.4% and interstitial pneumonia requiring immune-suppressive therapy. The externally mounted bovine pericardium of the bioprosthesis and tiny virtual valve to coronary distances measured with an anticipated diameter of 20 mm circle on pre-procedural multidetector computed tomography (MDCT) (Fig. 1a–g, ESM Video 1) suggested she was high-risk for CO with conventional TAVI. Considering the elevated frequency of stent thrombosis after TAVI with a self-expanding valve in failed surgical bioprosthesis [2], chimney stenting was an unfavorable strategy. Written informed consent and approval from the Institutional Review Board at our hospital was obtained to perform TAVI with BASILICA procedure.

**Fig. 1:**
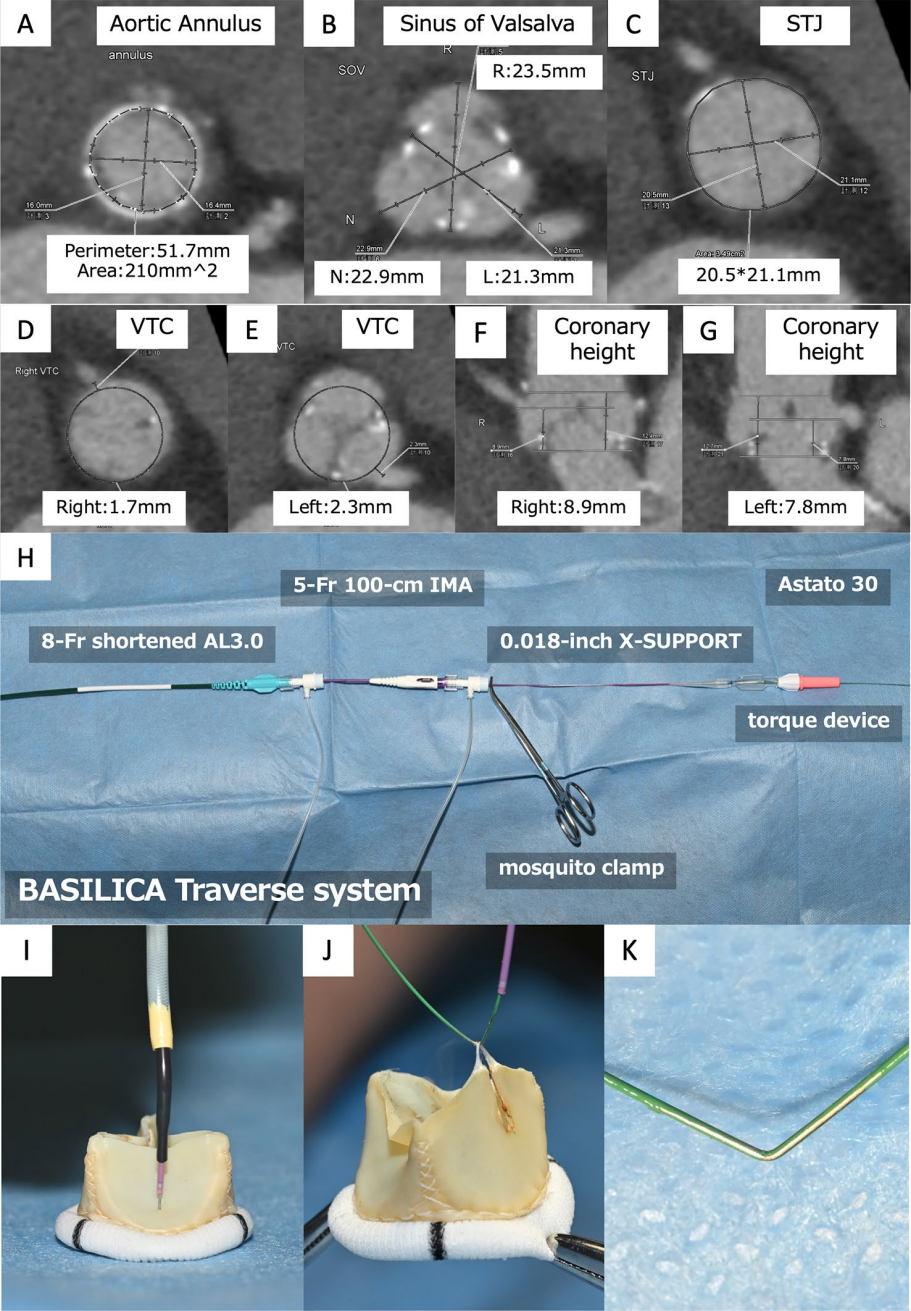
Pre-procedural measurement and pictures of in vitro experiment. **a**–**g** Pre-procedural measurements of multidetector computed tomography revealed a small bioprosthetic aortic annulus and tiny virtual transcatheter heart valve to coronary ostial distances. **h**–**k** Pictures taken in an in vitro experiment. **h** BASILICA leaflet traversal system. Catheters and a wire are fixed with a mosquito clamp and a torque device. **i** Distal configuration of the traversal system shows only the tip of the wire exposed to the leaflet. **j** Electrified wire was lacerating the leaflet accompanied by smoke and char formation. **k** Mid-portion of the Cruise wire was kinked and denuded only the inside surface with a scalpel blade

The procedure was performed under local anesthesia and sedation without transesophageal echocardiography (TEE) guidance. Although TEE guidance is generally recommended, we avoided it due to concern about the risk of acute exacerbation of interstitial pneumonia after positive pressure ventilation. We started the procedure with BASILICA on the left cusp first. We employed an 0.018-inch 135-cm X-SUPPORT microcatheter (ZEON MEDICAL) and an 0.018-inch 180-cm Astato 30 (ASAHI Intecc) for leaflet traversal. They were locked together with a mosquito clamp and torque device to prevent movement (Fig. 1h) instead of the built-in locking system of the PiggyBack microcatheter. The microcatheter was selected based on 0.018-inch compatibility, adequate working length, rigidness of the shaft and tip visibility under fluoroscopy. Confirming whole the traversal system is the optimal direction to the left cusp on both side and front views (Fig. 2a–d, ESM Video 2,3), the wire was electrified in pure cut mode at 100 W while gently advancing it (Figs. 1i, 2e, ESM Video 4). The wire passed through the left cusp and snared inside the left ventricle, followed by the microcatheter crossing through the leaflet (Fig. 2f). The wire was replaced with an 0.014-inch 300-cm Cruise (ASAHI Intecc) as an alternative for the Astato XS 20 300-cm guide wire. The laceration was successful by applying 100 W power to a “flying V” shaped Cruise wire (Figs. 1j, 2g, ESM Video 5). It was formed by scraping the wire with the non-sharp side of a scalpel to remove the insulating coating, then bending it inward (Fig. 1k). At the time, we decided not to attempt BASILICA on the right cusp because (1) we found a tiny space communicating the right and left Sinus of Valsalva on the pre-procedural MDCT (ESM Video 1). It suggested that BASILICA on the left cusp can maintain the right coronary flow, (2) already consuming much time, contrast media and X-ray. Furthermore, (3) side view of the right cusp is commonly not achievable due to severe angulation. Likewise, the pre-procedural evaluation showed an impossible right cusp side view fluoroscopic angle. Under the coronary protective system placed in the right coronary artery (RCA), the 23-mm Evolut pro plus (Medtronic) transcatheter heart valve was implanted. Subsequently, post-dilatation using an 18-mm Z-MED balloon (B. Braun Medical) was performed (Fig. 2h, ESM Video 6). The protective system was then retrieved after confirming the patent RCA (Fig. 2i). Final aortography showed optimal implant depth and patent both coronary arteries (Fig. 2j, ESM Video 7). In the final simultaneous left ventricular-aortic pressure measurement, the mean pressure gradient was 5.7 mmHg.

**Fig. 2:**
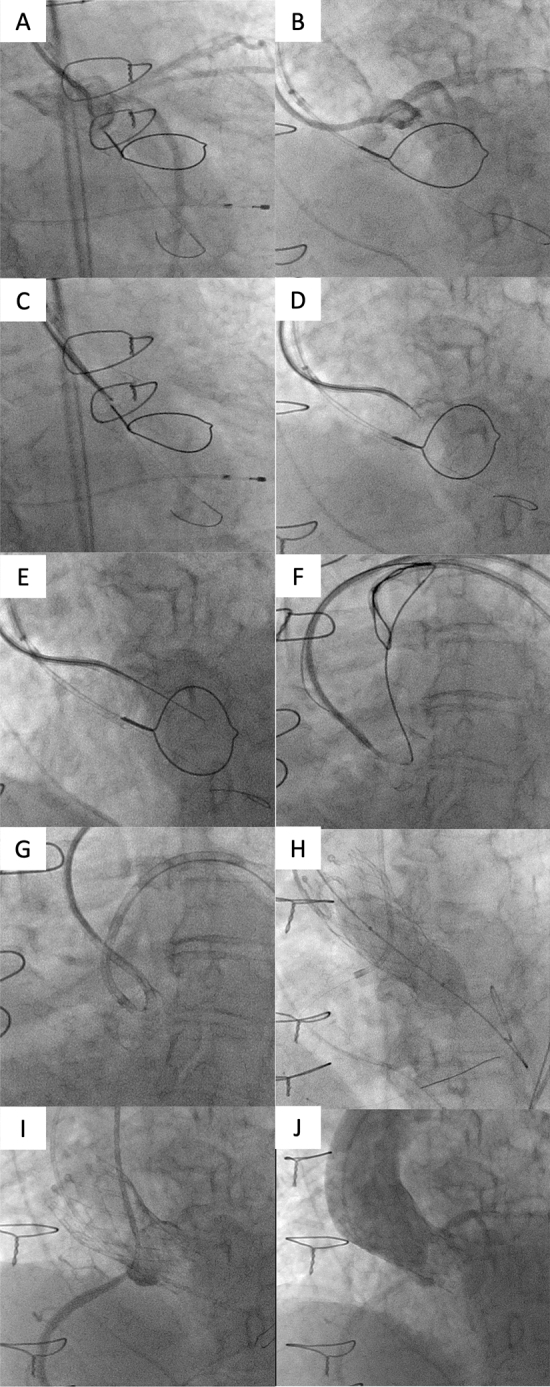
Intra-procedural fluoroscopic images. **a**–**d** Cusp injection of front view (**a**) and side view (**b**), traversal point of front view (**c**) and side view (**d**). **e** After traversal, the wire passed through the leaflet. **f** The microcatheter was also pulled up to the ascending aorta. **g** After exchanging the wire to a 300 cm Cruise wire, leaflet laceration was successful. **h** Post-dilatation with an 18-mm Z-MED balloon under the protection of the right coronary artery. **i**–**j** Injection from guiding catheter (**i**) and final aortography (**j**) confirmed patency of both coronary arteries and optimal implant depth

The post-procedural course was fair without myocardial injury and cerebral ischemia. The post-procedural transthoracic echocardiography showed a trans aortic peak pressure gradient of 3.34 m/s, mPG of 22 mmHg, effective orifice area of 1.07 cm^2^ and effective orifice area index of 0.71 cm^2^. The post-procedural MDCT revealed a separated left leaflet of Trifecta, no contrast enhancement of non-coronary cusp and a sealed Sino-tubular junction (Fig. 3, ESM Video 8).

**Fig. 3:**
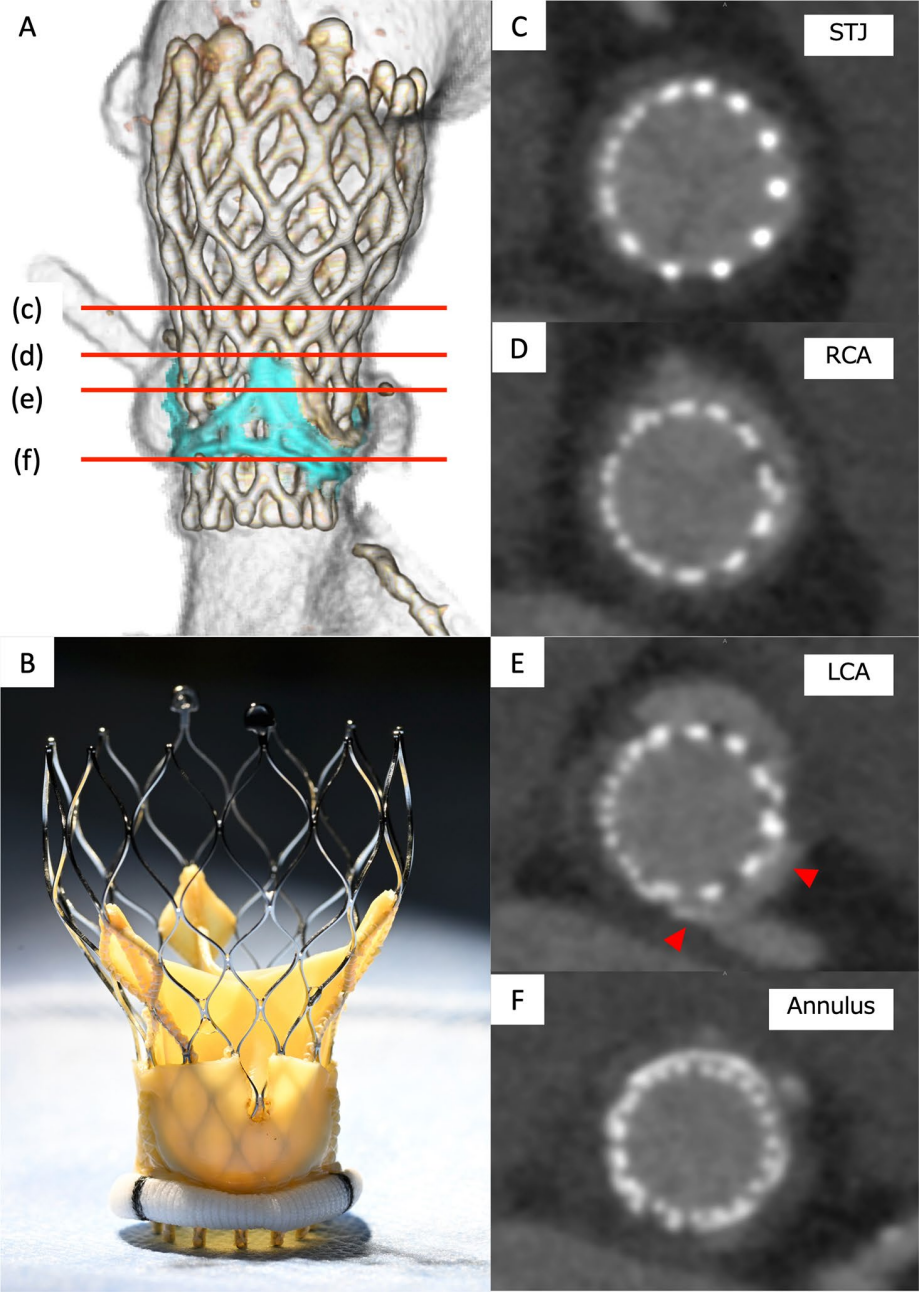
Post-procedural multidetector computed tomography images. **a** Volume-rendering image of the aortic root showing 23-mm Evolut pro plus (white) and 19-mm Trifecta (light blue). **b** Corresponding in vitro image demonstrating that a lacerated leaflet creates communication between the native left sinus of Valsalva and neo-sinus. **c** An axial image of the Sino-tubular junction shows the waist of the Evolut contacts circumferentially to the aorta. **d**, **e** Axial images at the level of right and left coronary arteries demonstrate contrast enhancement of both right and left sinuses of Valsalva, whereas non-coronary sinus is not enhanced. Inside the left cups, a calcified left leaflet of Trifecta separates on both sides (red arrow heads). **f** An annuls level image showed Evolut expanding to a circle with a diameter of 14.5 mm

We describe our experience with the first BASILICA procedure performed in Japan. Our case demonstrates the feasibility of the BASILICA procedure in a country where the required devices are unavailable.

## Supplementary Information

Below is the link to the electronic supplementary material.

Supplementary file1 ESM Video 1: Axial image of pre-procedural multidetector computed tomography image of the aortic root (MOV 21608 KB)

Supplementary file2 ESM Video 2: Front view of left cusp injection (MOV 12374 KB)

Supplementary file3 ESM Video 3: Side view of left cusp injection (MOV 16063 KB)

Supplementary file4 ESM Video 4: Successful traversal of the left leaflet of Trifecta (MOV 21298 KB)

Supplementary file5 ESM Video 5: Lacerating the left leaflet of Trifecta (MOV 48661 KB)

Supplementary file6 ESM Video 6: Post dilatation under the right coronary artery protection (MOV 37177 KB)

Supplementary file7 ESM Video 7: Final aortography (MOV 19522 KB)

Supplementary file8 ESM Video 8: Axial image of post-operative multidetector computed tomography image of the aortic root (MOV 13755 KB)

## Data Availability

Data sharing is not applicable to this article as no datasets were generated or analyzed during the publication.
